# Supplementary material for: PET metabolic tumor volume as a new prognostic factor in childhood rhabdomyosarcoma
Source: PLoS One. 2022 Jan 14;17(1):e0261565. doi: 10.1371/journal.pone.0261565 (PMC8759649; doi:10.1371/journal.pone.0261565)
Supplement: Attachment — Submitted filename: Response to Reviewers.docx [file pone.0261565.s002.docx]

**Rebuttal letter**

**Journal Requirements :**

**1.** Please ensure that your manuscript meets PLOS ONE's style requirements, including those for file naming.

We tried to do it well, following Plos ONE’s instructions.

**2.** You indicated that you had ethical approval for your study. In your Methods section, please ensure you have also stated whether you obtained consent from parents or guardians of the minors included in the study or whether the research ethics committee or IRB specifically waived the need for their consent.

According to the french law, the retrospectives non interventional studies do not require patient consent when the study protocol is compliant with the CNIL reference methodology repository about the retrospective data collection. In the case of an expressly written refusal, patient data were not analysed. This has allowed our work to be approved by the Ethics Committee of French Society of Nuclear Medicine and registered with the number CEMEN 2020-01. Thus, we could analyse PET images from patients and provide illustrative images.

**3.** Thank you for stating the following financial disclosure:

"The funders had no role in study design, data collection and analysis, decision to publish, or preparation of the manuscript."

At this time, please address the following queries:

**a)** Please clarify the sources of funding (financial or material support) for your study. List the grants or organizations that supported your study, including funding received from your institution.

Not concerned.

**b)** State what role the funders took in the study. If the funders had no role in your study, please state: “The funders had no role in study design, data collection and analysis, decision to publish, or preparation of the manuscript.”

Not concerned.

**c)** If any authors received a salary from any of your funders, please state which authors and which funders.

Not concerned.

**d)** If you did not receive any funding for this study, please state: “The authors received no specific funding for this work.”

Please include your amended statements within your cover letter; we will change the online submission form on your behalf.

It is now added in the cover letter**.**

**4.** In your Data Availability statement, you have not specified where the minimal data set underlying the results described in your manuscript can be found. PLOS defines a study's minimal data set as the underlying data used to reach the conclusions drawn in the manuscript and any additional data required to replicate the reported study findings in their entirety. All PLOS journals require that the minimal data set be made fully available. For more information about our data policy, please see http://journals.plos.org/plosone/s/data-availability.

"Upon re-submitting your revised manuscript, please upload your study’s minimal underlying data set as either Supporting Information files or to a stable, public repository and include the relevant URLs, DOIs, or accession numbers within your revised cover letter. For a list of acceptable repositories, please see http://journals.plos.org/plosone/s/data-availability#loc-recommended-repositories. Any potentially identifying patient information must be fully anonymized.

Important: If there are ethical or legal restrictions to sharing your data publicly, please explain these restrictions in detail. Please see our guidelines for more information on what we consider unacceptable restrictions to publicly sharing data: http://journals.plos.org/plosone/s/data-availability#loc-unacceptable-data-access-restrictions. Note that it is not acceptable for the authors to be the sole named individuals responsible for ensuring data access.

We will update your Data Availability statement to reflect the information you provide in your cover letter.

The data set is named data_set, in two files, Access and Excel. We removed the birth dates in order to not compromise confidentiality in the context of human-subject research. Date of diagnosis, relapse, death, still remain.

**5.** Your ethics statement should only appear in the Methods section of your manuscript. If your ethics statement is written in any section besides the Methods, please delete it from any other section.

It is ok.

6. Please review your reference list to ensure that it is complete and correct. If you have cited papers that have been retracted, please include the rationale for doing so in the manuscript text, or remove these references and replace them with relevant current references. Any changes to the reference list should be mentioned in the rebuttal letter that accompanies your revised manuscript. If you need to cite a retracted article, indicate the article’s retracted status in the References list and also include a citation and full reference for the retraction notice.

It is ok.

**Reviewers :**

**#1 :**

1. Please expand and provide examples on conflicting results on the prognostic utility of SUV.

*Added in the discussion :*

Most of PET studies use SUV, and especially SUVmax, to approach the tumor’s agressiveness, but with a certain variability of the measure (15,16). Thus Brendle et al. showed that SUV calculated on the same PET equipment acquisition was subject to variability according to the differents algorithms reconstructions, and that SUVmax was the least reproducible measurement comparing to SUVmean and SUVpeak (46). And the meta-analysis of Ghooshkhanei et al. in endometrial cancer illustrates this issue showing that three studies reported an association between the pre-operative SUVmax with disease free survival and/or overall survival. The HR of each study was calculated according to three differents cut off SUVmax (the values were 12.7, 17.7, and 8.35), highliting its variability, even more when PET machines are differents (47).

2. Explain also in your introduction the potential advantages of using MTV instead of SUV.

*This was added in the introduction complementary to the shorter justification of MTV choice already written:*

Single pixel values of the SUV, and especially SUVmax, are commonly used as a quantitative index of tumor metabolism, mainly because it is now well implemented on images viewers and thus easy to use. However this semiquantitative evaluation is subject to intra- and interindividual biases by a broad range of biological and technical factors such as patient’s weight, blood glucose level, acquisition parameters including uptake time, inaccurate calibration of PET, image reconstruction algorithm, etc. (15,16). To overcome these shortcomings, MTV approach, defined as the sum of the volume of voxels with SUV surpassing a threshold value in a tumor, can be considered (17). Recent studies confirmed the interest of MTV and sometimes its superiority compared to SUVmax with regard to prognostic value, in various neoplastic pathologies such as Hodgkin's lymphoma, ovarian squamous cell carcinoma, non-small cell lung cancer, metastatic colorectal cancer, and pancreatic cancer (18–23).

3. Explain the choice of 40% threshold for MTV.

*This was added in the Methods and Materials part in PET protocol, complementary to the shorter justification already written:*

From the physics side, after phantom studies (28,29), a threshold value of 40% to define the tumor boundary on PET images was used in many clinical studies (30–33). This 40% threshold is the most common index in clinical practice for evaluating tumor prognosis (34,35). Nowadays PET imaging softwares offer an automatic 40% SUV approach to delineate tumor contours. Thereby a threshold of 40% of the SUVmax was applied in our study.

**#2 :**

There are some wrong punctuation in the paper. Please check and edit.

**1-**In the introduction:

-Please add the lung CT scan as part of the initial staging exams.

The lung CT scan as a part of the initial staging exams was added:

“After initial clinical symptoms, such as a swelling, paraclinical tests [...] lesion biopsy, local imaging (lung-CT, MRI), lumbar puncture […] metastatic lesions (5).”

-The paragraph: "identification of unfavorable factors such as age ..." is not clear. Please distinguish the prognosis of localized patients without mentioning the distant metastatic tumor spread, compared to patients with metastatic disease.

-In the sentence "...leading to a cure rate close to 70% in RMS sites": please change "RMS sites" with "localized RMS"

The manuscript has been modified :

Indeed, the global cure rate for RMS (for all risks groups) has improved from 25–30% to approximately 70%, in the 30 last years.

“Identification of unfavourable factors such as age (> 10 years), alveolar histological subtype, size (> 5 cm) and location of the primary tumour (parameningeal, limbs, or trunk), and presence of regional nodal or distant metastatic tumour spread sites, has allowed a risk classification to optimize the treatment the combination of chemotherapy and surgery to be optimized, leading to an improvement of the cure rate from 25–30% to approximately 70% (6). Thus patients in the low risk group have the best prognosis progression-free survival (PFS) and overall survival (OS) (3-year PFS rate of 88%) (7). However, patients with metastatic disease still have a dismal prognosis (OS: 0‑30%) (8)..”

-”PET has been shown to be superior to CT for the initial staging of RMS”. This sentence is not completely true. Some studies (of which the most recent and extensive by Mercolini et al., EJC) demonstrated a superiority of PET compared to CT in some sites but not in others (eg intrathoracic sites). Please edit the sentence and add a reference to the bibliography

The sentence has been modified :

“Apart from detecting intrathoracic lesions where chest CT remains essential, F-18 FDG PET-CT (PET) has been shown to be superior to CT for the initial staging of RMS, mainly on account of its ability to detect nodal involvement and metastatic disease (9)”

-Please use “nodal” instead of “ganglion”

The term has been changed.

**2-** In the “patients population” paragraph:

-The sentence “patients who had undergone a PET-CT after excision of the primary tumor …, were exluded” in my opinion it is not well written and needs to be clarified

The sentence has been modified :

“Patients who had undergone a PET-CT examination while the primary tumor was already excised by surgery, who had commenced chemotherapy, who had RMS located in the bladder or in a parameningeal site (where measuring MTV was impossible, owing to the close physiological activity of the bladder or brain), whom PET images where unrecorded, or who had a tumour in a limb that was not within the scope of acquisition, were excluded.”

**3-** In the “Treatment” paragraph:

-The sentence “The monitoring methods after the end of the treatment were also defined by the protocol” is repeated 2 times. Please remove 1 of the two.

The repeated sentence is deleted.

**4-** In the “Statistical analysis” paragraph:

- please remove the first sentence ("Because RMS is a rare disease, several centers .... sample size population")

The first sentence was deleted

**5-** In the discussion:

- “The age distribution is as reported as previous study with a peak around young infancy”. The reference 29 is not a study and in my opinion the sentence is not clear.

The sentence and the reference have been modified :

“The age distribution is as previously described with a bimodal age peaks in childhood (40).”

- Please edit.

"in a previous study of 108 patients combining MRI and CT scans, Ferrari et al .....". Please specify that these are patients with rhabdomyosarcoma.

It is added.
